# Probing the SELEX Process with Next-Generation Sequencing

**DOI:** 10.1371/journal.pone.0029604

**Published:** 2011-12-29

**Authors:** Tatjana Schütze, Barbara Wilhelm, Nicole Greiner, Hannsjörg Braun, Franziska Peter, Mario Mörl, Volker A. Erdmann, Hans Lehrach, Zoltán Konthur, Marcus Menger, Peter F. Arndt, Jörn Glökler

**Affiliations:** 1 Department of Vertebrate Genomics, Max Planck Institute for Molecular Genetics, Berlin, Germany; 2 Institute for Chemistry/Biochemistry, Free University Berlin, Berlin, Germany; 3 Department of Computational Molecular Biology, Max Planck Institute for Molecular Genetics, Berlin, Germany; 4 Alacris Theranostics GmbH, Berlin, Germany; 5 Institute of Biochemistry, Universität Leipzig, Leipzig, Germany; 6 RiNA RNA-Netzwerk Technologien GmbH, Berlin, Germany; Deutsches Krebsforschungszentrum, Germany

## Abstract

**Background:**

SELEX is an iterative process in which highly diverse synthetic nucleic acid libraries are selected over many rounds to finally identify aptamers with desired properties. However, little is understood as how binders are enriched during the selection course. Next-generation sequencing offers the opportunity to open the black box and observe a large part of the population dynamics during the selection process.

**Methodology:**

We have performed a semi-automated SELEX procedure on the model target streptavidin starting with a synthetic DNA oligonucleotide library and compared results obtained by the conventional analysis *via* cloning and Sanger sequencing with next-generation sequencing. In order to follow the population dynamics during the selection, pools from all selection rounds were barcoded and sequenced in parallel.

**Conclusions:**

High affinity aptamers can be readily identified simply by copy number enrichment in the first selection rounds. Based on our results, we suggest a new selection scheme that avoids a high number of iterative selection rounds while reducing time, PCR bias, and artifacts.

## Introduction

**Figure 1:**
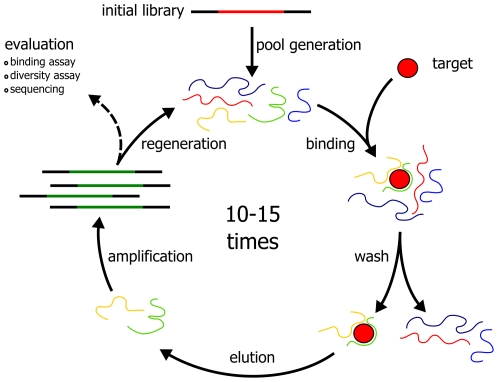
Schematic outline of the general SELEX procedure.

SELEX (“**S**ystematic **E**volution of **L**igands by **Ex**ponential Enrichment”) has been introduced independently in the labs of Larry Gold [1] and Jack Szostak [2] as a novel *in vitro* selection technique for the generation of high affinity nucleic acid binders (termed aptamers) to almost any given target. It generally involves many repetitive selection steps, followed by an analysis of enriched nucleic acids (see Figure 1). Many improvements to the original SELEX protocol have been made over the last two decades. Most of these address the mode of selection, immobilization, stringency and automation [3]. However, the advent of next generation sequencing technologies had little impact on aptamer selections so far. Performing many selection rounds on a target is time consuming and cumbersome if performed manually. Few publications can be found that describe the multiple problems and pitfalls that are associated with SELEX [4]–[6], such as PCR artifacts, background binders and targets that are unsuited as nucleic acid ligands. Due to the SELEX procedure, the nucleic acid population can be compared to a black box that remains closed until the last selection round. It is finally opened by sequencing of some hundred clones with the inherent risk of identifying only unspecific artifacts. We have investigated the fluctuation of library diversities during SELEX by analyzing re-melting profiles of DNA pools before [7]. However, enrichment of individual sequences that may exist in extremely low copies in the initial selection rounds can not be detected by this assay alone. Here, we show that next generation sequencing allows a successful isolation of high quality target binders after very few selection rounds, leading to a dramatic improvement in terms of duration and amount of experimental procedures necessary. First examples that demonstrate the usefulness of next-generation sequencing in library selections have been published [8]–[10]. To this end we have analyzed the selection of aptamers using Illumina's high-throughput sequencing platform on pools from all selection rounds. As a model target, we have used streptavidin immobilized on magnetic beads. Following an automated selection protocol that we have developed earlier [11], ten selection rounds were performed and the results were compared with those obtained by conventional Sanger sequencing.

## Results

### Semi-automated Selection

Starting with a synthetic oligonucleotide library with 40 randomized bases, we have performed ten selection rounds. In each round different means of stringency were applied in order to enrich aptamers specific for streptavidin. In total, we have decreased the amount of input DNA, target molecule concentration and incubation times and increased the number of washing steps as well as washing volumes (Table 1). The resulting DNA yields after PCR and purification were quantified to monitor the enrichment of binders. In order to reduce PCR artifacts and to keep the library free from aberrantly replicating sequences, we have performed as little PCR cycles as necessary.

**Table 1:** SELEX protocol.

Round	Input DNA [µg]	dsDNA [pmol]	Streptavidin-Beads [µg]	Streptavidin [pmol]	Incubation time [min]	Washing Steps
1	50	2000	1000	70	50	1×500 µl
2	3	120	500	35	50	1×500 µl
3	2	80	500	35	50	2×500 µl
4	0,8	32	500	35	50	2×500 µl
5	0,2	8	500	35	50	2×1000 µl
6	1,4	56	500	35	50	2×1000 µl
7	2,8	112	500	35	50	2×1000 µl
8	3	120	500	35	50	2×1000 µl
9	2	80	500	35	26	2×1000 µl
10	2	80	200	14	26	2×1000 µl

### Diversity assay

**Figure 2:**
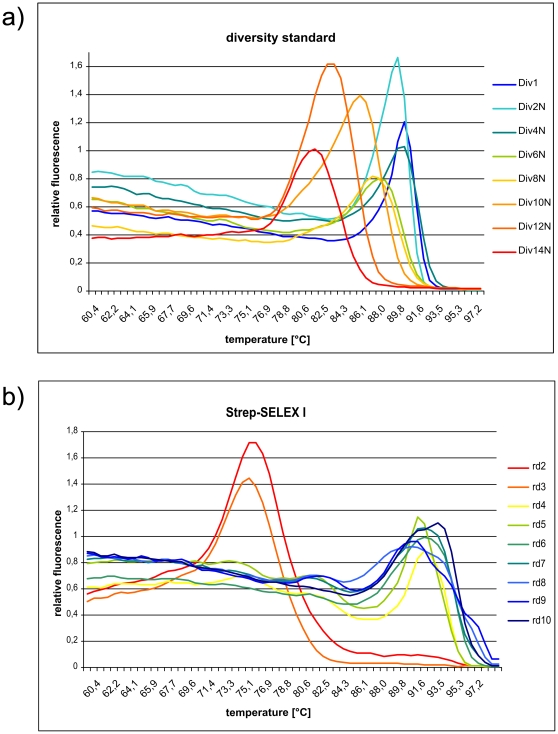
Remelting curves of diversity assay. a) diversity standard and b) selection rounds (1–10).

The enrichment of target-specific sequences and thus the loss of diversity over the course of selection could be monitored effectively with our diversity assay [7]. Following the principle of the C_o_t analysis [12], the diversity assay is based on the remelting kinetics of double-stranded DNA pools. Generally, the more diverse the DNA pool the longer it will take to re-hybridize after complete denaturation. Synthetic standard oligonucleotides with defined diversities allow a direct comparison with unknown pools. Remelting profiles (Figure 2) clearly indicate the collapse of diversity after the third round. Comparison of remelting temperature to the diversity standard indicates that from round 4 on, the population consists of less than 10^3^ different sequences and kept constant until the end of selection.

### Next Generation Sequencing results

We have obtained a total number of about 13 million reads by Illumina sequencing, of which 56% could be sorted according to their barcodes (see Table S1). A further screening for inserts of 40 bases with a tolerance of two bases resulted in 43% or 5.5 million reads that were used for our analysis. Despite equimolar mixture of barcoded and purified PCR products for the sequencing pool, the identified barcodes were not evenly distributed. The resulting pool of selection round 1 (barcode S1A02) did account for 1.6% of total reads in comparison to the most abundant reads in the unselected pool (barcode S1A01) of 5.3%. The barcoded sequences with proper insert length were finally sorted by copy number and a compiled file containing lists of the 100 most frequent clones of all ten selection rounds including the initial pool is available in supplemental data 1–11 (tabs round 0–10). Eight clones including some of the most enriched sequences were tracked over the selection rounds and are shown in Table 2 and were later studied in greater detail.

**Table 2:** List of oligonucleotides identified by Illumina sequencing after 10 selection rounds and analyzed in this study with parts corresponding to the putative consensus sequences printed in bold.

Clone	Sequence	Percentage in the 10th selection round
R10#1	5′-TCTTCCCATTCGGAGGCCTGCGCCGGCCGCGTCCGAGGGT	10%
R10#1a	5′-ACCCTCGG**ACGC**GGCCGG**CGCA**GGCCTCCGAATGGGAAGA	
R10#2	5′-GATTGCCCCTCG**ACGC**AGAGGT**C**TG**A**GTTGGTACAAATTA	6%
R10#4	5′-C**ACGC**GACCGG**CGCA**GGTCTGAGGGCAGGTCCAGTAATAT	5%
R10#6	5′-**ACGC**GCCGAT**CGCA**GGCTAGAGCTAATGGCTCAGGTTTAT	3%
R10#10	5′-ACTTGGACCCG**A**T**GC**CCGTGG**CGCA**ACGGTCGGGTCCTTC	1%
R10#17	5′-ATCTCCGATTGCCCCACG**ACGC**AGTGGT**CG**G**A**GTTACTTT	0.7%
R10#62	5′-GAGCATGGGGT**ACGC**ACCGAT**CGCA**GGTTAGCCCTAAAGT	0.1%
R10#86	5′-ATCTCCGATTGCCCCACG**ACGC**AGTGGT**CG**G**A**GATACTTT	0.1%

### Fluorescent dye-linked aptamer assay (FLAA) results

**Figure 3:**
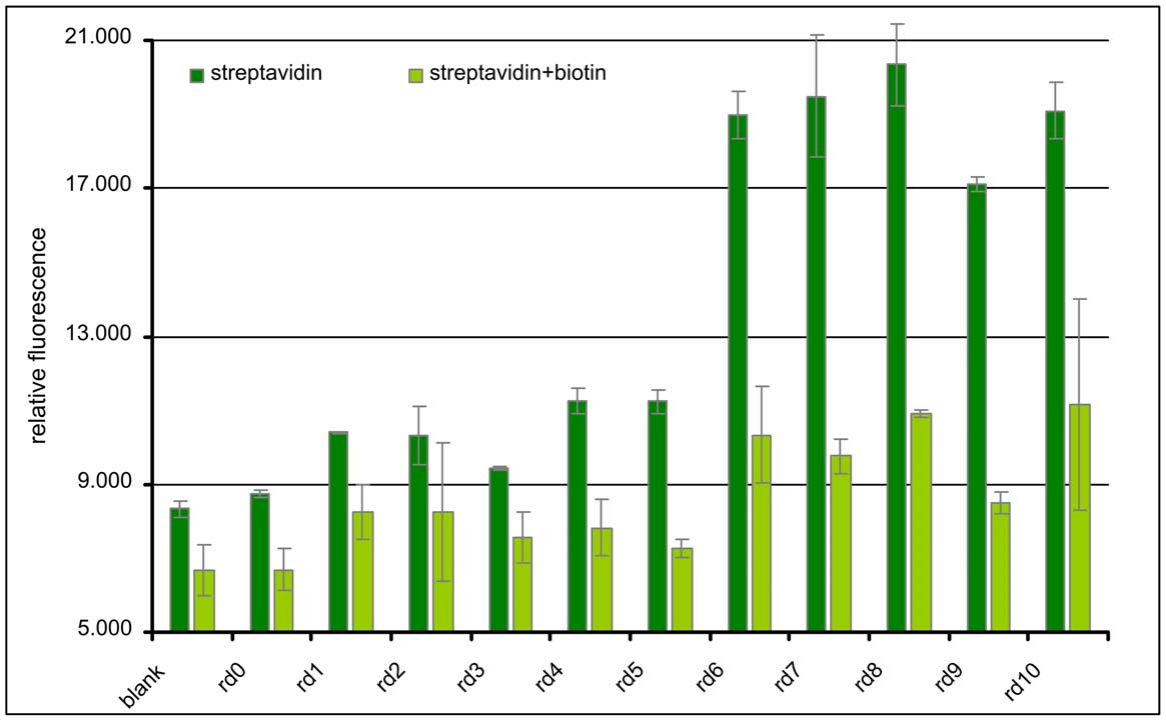
Microtiter plate binding assay (FLAA) of selection pools to immobilized streptavidin. Depicted are rounds 1 to 10 with the original library pool indicated as round 0.

**Figure 4:**
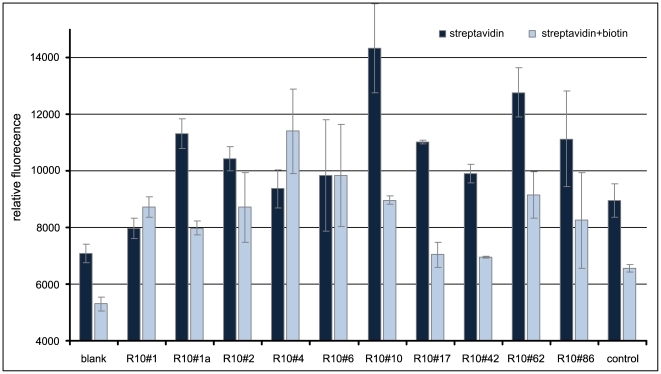
Microtiter plate binding assay (FLAA) of single clones applied as synthetic oligonucleotides to immobilized streptavidin.

The enrichment of binding sequences by the selection procedure (round 0 to 10) could be demonstrated by the non-radioactive FLAA (Figure 3). Additionally, synthetic oligonucleotides of selected single clones that were identified by Sanger and high-throughput sequencing were tested for their binding performance. Observed binding capacity was high for the clones R10#10 and R10#62 and slightly lower for R10#17, R10#86 and R10#1a (Figure 4). Unexpectedly, some of the selected clones displayed an elevated background signal on biotin-blocked streptavidin.

### Surface plasmon resonance (SPR) results

**Figure 5:**
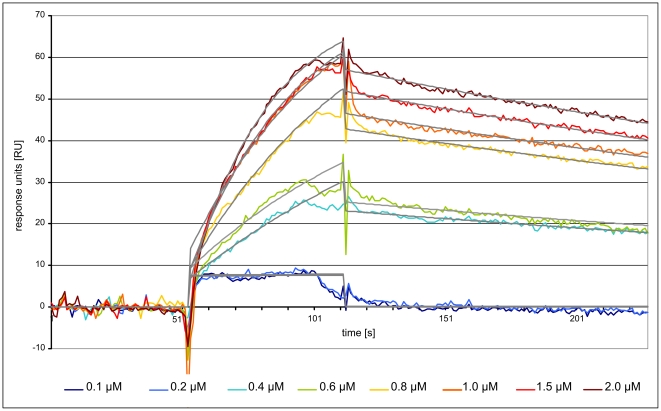
SPR sensorgram of streptavidin-aptamer binding. Streptavidin was covalently immobilized on a CM5-chip. Aptamer clone R10#17 with highest affinity determined by FLAA was injected at a concentrations ranging between 0.1 and 2 µM in binding buffer.

Due to the dense surface coating of the commercially available streptavidin chip, the effect of rebinding made the measurement and especially the evaluation of binding kinetics difficult. For this reason, we have coupled a chip with a lower density of streptavidin (900 RU) to allow a better study of the binding kinetics. SPR sensorgrams for the best binding aptamer R10#17 in dilutions of 100 nM to 2 µM are shown in Figure 5. Based on association rate constant (k_on_) of 1.5×10^4^ M^−1^ s^−1^ and dissociation rate constant (k_off_) of 2.2×10^−3^ s^−1^ a K_d_ value on 140 nM was obtained after global fitting to a 1∶1 Langmuir binding model. The ***χ***2 value, a standard statistical measure of the closeness of fit, was 0.535.

**Figure 6:**
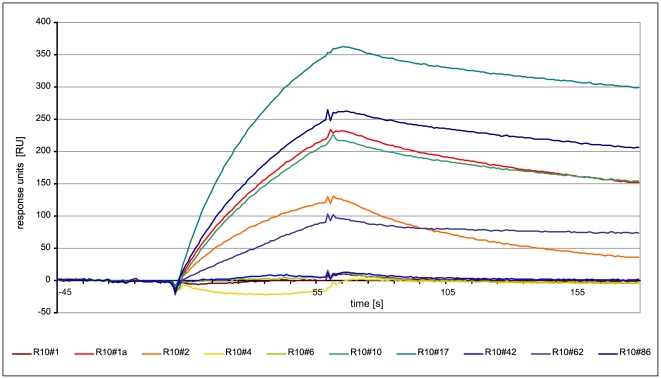
SPR sensorgram of selected oligonucleotides for direct comparison of binding affinity at identical concentration (1 µM).

Analogous to the measurement of binding constants, all identified FLAA-tested oligonucleotides were used in further SPR experiments. 1 µM aptamer solutions were analyzed on a second chip with immobilized streptavidin (7000 RU). The corresponding sensorgrams are shown in Figure 6. The color code indicates the ranking position of the clone in the 10^th^ selection round (red: high enrichment, blue: low enrichment). Best binding results were observed for the clones R10#17 and R10#86. The most frequent clone R10#1a showed affinity for the target in “antisense” direction, not in “sense” orientation. Additionally, clone R10#62a displayed a very low dissociation kinetic, different from all other tested clones. Strong background clones identified in the FLAA were negative in the surface plasmon resonance experiment.

## Discussion

**Figure 7:**
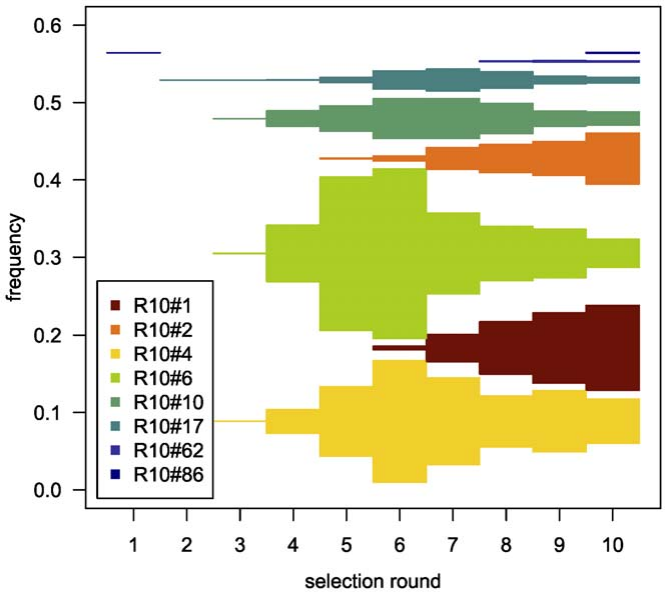
Relative frequency of clones in the pools (top100) of round 1 to 10 as evaluated by next generation sequencing.

**Figure 8:**
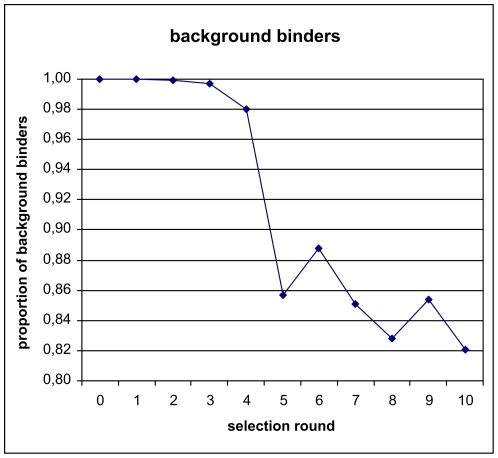
Loss of background binders characterized as unique clones in sequenced pools over the entire selection procedure.

Based on a semi-automated approach for the selection of DNA aptamers [11], we have adapted our original procedures to work on a robotic magnetic particle processor designed to work in a standard microtiter plate format. Generally, such high-throughput automation allows the application of reproducible conditions while still retaining greatest flexibility [13]. The synthetic DNA library was designed to contain a stretch of 40 randomized bases flanked by 18 constant bases as primer binding sites for PCR amplification. The primer sites were checked to be devoid of hairpin, dimer, and heterodimer-forming sequences. In order to remain flexible in terms of next generation sequencing platform for analysis, we did not directly adopt Illumina's primer sequences. We have omitted the generation of single-stranded DNA by the popular biotin-streptavidin method because of the target protein is already Streptavidin itself. However, the successful use of double-stranded DNA in SELEX has been demonstrated before [14], most likely because highly diverse DNA pools remain single stranded after denaturation [7]. In order to follow the selection process, we have monitored the PCR yield of every selection round analyzed the pools for diversity [7] and observed binding behavior by a fluorescent dye-linked aptamer assay (FLAA) [15]. Despite the increased stringency in the course of the selection, a strong increase yield of PCR product was observed (see Table S2). The diversity assay also shows a dramatic drop of pool diversity following the third selection round (see Figure 2). However, an efficient target binding was not detected by FLAA until round four. The binding signal then increased strongly until round six. We have sequenced 25 clones by conventional Sanger sequencing after the 10^th^ selection round, albeit with deletions and missing insert (see Table S3). However, we could identify several sequences associated with specific binding by FLAA. We were interested in the first appearance of specific binders and their dynamics during the selection and performed next generation sequencing of library pools from all ten selection rounds. For analysis, we have compiled lists containing the top 100 occurring sequences (see Supporting Information S1). Interestingly, the best binders (R10#17 and R10#86) that were identified by Sanger sequencing of the tenth selection round already exist in the very first round, albeit at a very low copy number (7 and 4 respectively of 2×10^6^ total reads) just above the background of about two copies per sequence. Enriched binders became more apparent in the second and third selection round. In the fourth round, in which binding could be detected by FLAA, the most frequent clone already accounts for 11% of all sequencing reads. The highest count (22%) of a single clone is observed in round six (Figure 7). The number of low-frequency background binders, characterized by single sequences, drops sharply after round four (Figure 8) and levels off to almost 80%. However, most or the unique clones that occur after round 5 are derivatives of strongly enriched clones which can be attributed to either mutation or sequencing artifacts.

**Figure 9:**
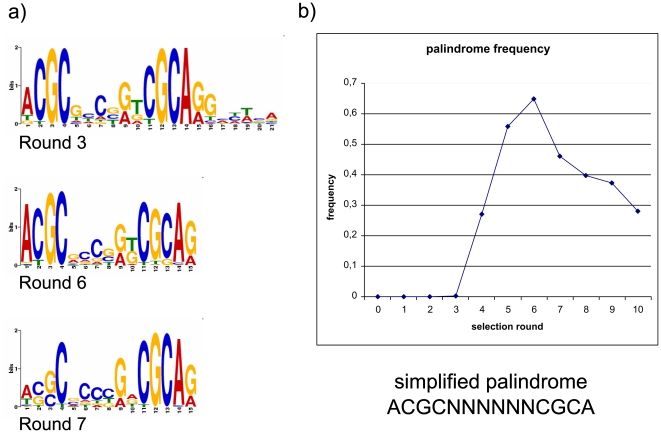
Sequence motifs and distribution. a) Motifs identified by MEME from some selection rounds. b) Frequency of simplified palindrome in the sequence pools (top100) of round 1 to 10.

The most prominent motif for streptavidin binding was identified by MEME [16]. Since MEME is not designed to process more than 1000 clones, we decided to select only the topmost enriched sequences (max. 800 clones) for analysis. We identified a motif in the third selection round that only gets slightly more defined in later selection rounds (Figure 9a). However, the palindrome of ACGCNNNNNNCGCA identified from the 3^rd^ round fits most sequences that are enriched during the selection rounds on Streptavidin (Figure 9b). Generally, these motifs are similar to DNA aptamers selected against streptavidin found in other studies [17]–[19], which have been compared and analyzed in more detail by Bing *et al.* [20]. It seems that these are of more structural relevance [20] and thus difficult to discover by word-based methods.

The constant primer sequences of the library have been designed not to form any secondary structure and should not strongly interfere with those in the randomized area of the library. Because the clones identified by high-throughput sequencing do not exist in an isolated physical form as the obtained Sanger clones, we decided to order fully synthetic oligonucleotides encompassing only the variable part of 40 bases without the constant regions for further binding analysis. Since the FLAA analysis can only be used as a qualitative binding assay, surface plasmon resonance was used for quantitative analysis of the selected aptamer binding properties.

**Figure 10:**
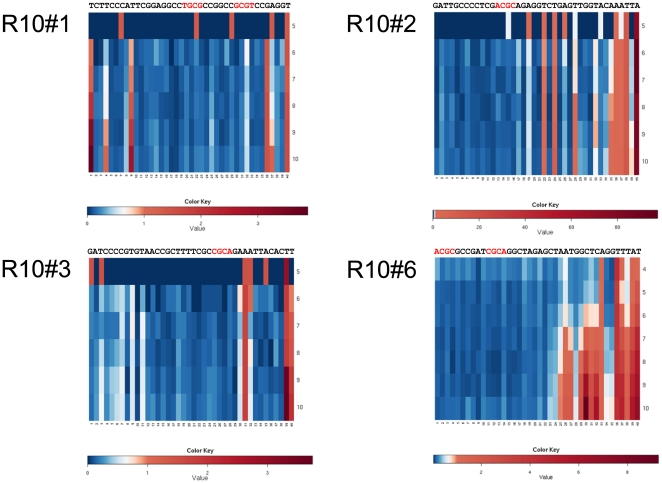
Heat map indicating mutation frequency in selected abundant clones over selection rounds 5 to10 listed in successive rows. Red color represents high mutation rates in the respective positions whereas little ore no mutations are colored blue.

An important issue of performing many selection rounds is the introduction of mutations by Taq polymerase. Indeed, we have observed mutant clones to arise during the course of selection which reflects the use of Taq polymerase for turning SELEX into an *in vitro* evolution method if many selection rounds are performed. Some of the abundant clones were found to accumulate mutations over the selection rounds (Figure 10). Mutations occur mainly outside the proposed consensus motifs. However, one such substitution in the enriched clone R10#17 over R10#86 did change the binding affinity in our SPR experiments and proved to be the strongest binder that we have analyzed (Figure 5 and 6).

Interestingly, the most abundant clones analyzed in the final selection round did not directly coincide with the strongest binding behavior. However, some of the enriched clones may bind only in the context of primer sequences or in the antisense orientation (R10#1a). The best binders were found to accumulate very early in the first (R10#86) and second selection rounds (R10#17). This distribution indicates a strong selection bias in favor of PCR performance over binding properties.

Based on our results and in accord with other new developments [21] we recommend to perform three selection rounds on a given target immobilized on magnetic beads and sequence the pools by a next generation method. Scoring the copy numbers of certain clones will help identifying signatures of specific binders that become obvious in the later selection rounds. Those signatures help to pick those clones that already appear in the first selection rounds and are thus more likely belonging to the set of better binders. A simple analysis by FLAA helps identifying the strong binders that can be analyzed in further detail by SPR. This approach may allow to shorten SELEX experiments including the more lengthy and cumbersome RNA selections [13], and to retrieve high affinity binders from highly diverse random libraries.

## Materials and Methods

### Materials

All oligonucleotides including the DNA library of 40 randomized bases (Bank40) were purchased from Purimex RNA/DNA Oligonucleotides (Grebenstein, Germany). Semi-automated selections were performed with Magnetic Particle Processors Kingfisher Flex (Thermo Scientific, Bonn, Germany). Streptavidin magnetic beads (Dynabeads® M-280 Streptavidin) were purchased from Invitrogen GmbH (Karlsruhe, Germany). Taq DNA Polymerase (EURx, Poland) was used for amplification after each selection round and resulting products were purified with sbeadex® (LGC Genomics, Germany).

### Semiautomatic *in vitro* selection

2 nmol of the Bank40 library was initially amplified by PCR for 3 cycles (95°C for 30 s, 95°C for 2 min, 55°C for 1 min, 72°C for 3 min, 3 cycles, 72°C for 5 min). Input for the first selection round was 2 nmol of dsDNA purified from the PCR reaction (Bank40), denatured (94°C for 3 min) in binding buffer (1×) and chilled on ice. Binding and washing steps were performed in 1× binding buffer (20 mM Tris, 140 mM NaCl, 5 mM MgCl_2_, 2 mM CaCl_2_, 2 mM KCl and 0.05% Tween 20 (pH 7.4)). Streptavidin aptamers were generated by SELEX protocol outlined in Table 1 using streptavidin-coated magnetic beads and the KingFisher Flex Magnetic Particle Processor (Thermo Scientific). PCR for amplification of retained DNA was performed directly on magnetic beads without elution step.

### Cloning and Sanger sequencing

PCR products of the 10^th^ selection round were inserted into pCR®2.1-TOPO by the use of TOPO® TA Cloning® Kit (Invitrogen) according to the manufacturer's instructions and *E. coli* cells (DH5α) were transformed via heat shock. Overnight cultures were grown from single colonies and plasmid DNA was extracted and purified by NucleoSpin® Plasmid (Macherey-Nagel, Düren, Germany). Plasmid inserts of the selected clones were sequenced by conventional Sanger method.

### DNA pool generation for Illumina sequencing

Barcodes were attached by PCR to the pools of all selection rounds. The samples were amplified using the SPro5 forward primer and the SPro3 index primers containing 6mer barcodes suggested by Illumina Inc. (SiA1Pro3-SiA11Pro3, Table S4). After purification of the samples using Wizard SV Gel and PCR Clean-Up System (Promega Corporation, USA) all samples were mixed in equimolar amounts (each 500 ng) and sequenced in a single lane of an Illumina Genome Analyzer GA2 for 100 bases.

### Sequence data evaluation

Base calling following the sequencing procedure was done with the Bustard package. In total nearly 13 million reads were obtained. Subsequently primers and tags specific for each selection round were mapped to each read using RazerS [22]. A list of tags and primers can be found in Table S4. To reduce analysis of artifacts, only those reads were kept that mapped without mismatches and had a random part with a length between 38 and 42 bp. About 5.5 million clones (43%) fulfilled these criteria and could be assigned to one of the rounds depending on their tag.

### Qualitative binding studies by fluorescent dye-linked aptamer assay (FLAA)

The enrichment of binding sequences over the course of selection was monitored with a non-radioactive fluorescence microtiter plate assay [15]. Streptavidin coated 96 well microtiter plates were incubated with 12 pmol of dsDNA of the initial library stock in 50 µl selection binding buffer for 1 h at room temperature. In the analysis of single clones, 12 pmol synthetic ssDNA oligonucleotides were applied per well under identical conditions. Biotin-blocked wells were used as negative controls. Wells were washed twice with 200 µl binding buffer before 50 µl OliGreen (1∶500 in binding buffer, Molecular Probes®, Eugene, USA) was added and after incubation of 9 min relative fluorescence (excitation 485 nm, emission 527 nm) was measured (POLARstar Omega, BMG Labtech).

### Affinity determination by surface plasmon resonance

The Biacore™ X platform (GE Healthcare, USA) was used to perform binding analysis of the selected ssDNA aptamers. Therefore, Streptavidin was immobilized onto a CM5 sensor chip (GE Healthcare, USA) utilizing amine coupling. Immobilization was carried out at a flow rate of 5 µl/min in 20 mM HEPES, 150 mM NaCl, 3 mM EDTA and 0.005% Surfactant P20 (pH 7.4). The surface of the CM5 chip was activated for 7 min with 0.02 M EDC (1-ethyl-3-(3-dimethylaminopropyl)-carbodiimide) and 0.05 M NHS (N-hydroxysuccinimide). After the injection of 10 µl 10 µg/ml Streptavidin (ImmunoPure® Streptavidin, Thermo Scientific, Bonn, Germany) in 10 mM sodium acetate (pH 4), 35 µl of 1 M ethanolamine (pH 8.5) were used to block remaining reactive groups followed by two washing steps. The procedure resulted in an increase of ca. 900 response units (RU) on channel 2. A reference cell was generated by activating and blocking without adding streptavidin (channel 1). Binding analysis was conducted at a flow rate of 30 µl/min with binding buffer at 25°C. Prior to injection, synthetic ssDNA oligonucleotides were denatured for 3 min at 94°C and refolded in binding buffer. 30 µl of the ssDNA solution in a range from 0.1 to 2.0 µM were injected into both channels of the flow cell. After each ssDNA injection, the chip surface was regenerated by injection of 2×10 µl 0.5 mM NaCl/0.5 mM MgCl_2_. For background correction, the response signal of the reference cell was subtracted from the signal of the immobilized surface.

Association and dissociation rates and constants of the aptamer–streptavidin complexes were determined using BIAevaluation software (version 4.1, Biacore).

## Supporting Information

Supporting Information S1 Top100 of Illumina-sequenced library pool (Bank40).
(XLS)

Table S1 Efficiency of Illumina sequencing and barcoding. Insert refers to reads of expected length of approximately 40 bases.
(DOC)

Table S2 PCR yields from selection-round amplification.
(DOC)

Table S3 list of clones, sequenced by Sanger method.
(DOC)

Table S4 list of oligonucleotides used in this study.
(DOC)
