# Supplementary material for: Probing the SELEX Process with Next-Generation Sequencing
Source: PLoS One. 2011 Dec 29;6(12):e29604. doi: 10.1371/journal.pone.0029604 (PMC3248438; doi:10.1371/journal.pone.0029604)
Supplement: Table S1 — Efficiency of Illumina sequencing and barcoding. Insert refers to reads of expected length of approximately 40 bases. (DOC) [file pone.0029604.s002.doc]

Supporting Table 1. Efficiency of Illumina sequencing and barcoding. Insert refers to reads of expected length of approximately 40 bases.

| **Sequences** | **Selection round** | **count** | **%** | **insert** | **% insert** |
| --- | --- | --- | --- | --- | --- |
| total |  | 12,852,327 | 100.00 | NA | NA |
| barcoded |  | 7,219,413 | 56.17 | 5,538,281 | 43.09 |
| S1A01 | (Bank40) 0 | 723,183 | 5.63 | 678,306 | 5.28 |
| S1A02 | 1 | 221,245 | 1.72 | 204,388 | 1.59 |
| S1A03 | 2 | 1,004,467 | 7.82 | 896,827 | 6.98 |
| S1A04 | 3 | 753,614 | 5.86 | 641,239 | 4.99 |
| S1A05 | 4 | 599,471 | 4.66 | 542,646 | 4.22 |
| S1A06 | 5 | 319,493 | 2.49 | 280,205 | 2.18 |
| S1A07 | 6 | 722,320 | 5.62 | 605,841 | 4.71 |
| S1A08 | 7 | 711,308 | 5.53 | 637,870 | 4.96 |
| S1A09 | 8 | 307,193 | 2.39 | 270,572 | 2.11 |
| S1A10 | 9 | 554,912 | 4.32 | 495,132 | 3.85 |
| S1A11 | 10 | 435,968 | 3.39 | 285,255 | 2.22 |
