# Supplementary material for: Probing the SELEX Process with Next-Generation Sequencing
Source: PLoS One. 2011 Dec 29;6(12):e29604. doi: 10.1371/journal.pone.0029604 (PMC3248438; doi:10.1371/journal.pone.0029604)
Supplement: Table S2 — PCR yields from selection-round amplification. (DOC) [file pone.0029604.s003.doc]

Supporting Table 2. PCR yields from selection-round amplification

| **Round** | **PCR Cycles** | **PCR-Product [ng]** |
| --- | --- | --- |
| 1 | 15 | 7670 |
| 2 | 15 | 2565 |
| 3 | 15 | 1775 |
| 4 | 15 | 720 |
| 5 | 15 | 2680 |
| 6 | 14 | 3795 |
| 7 | 12 | 4335 |
| 8 | 10 | 3745 |
| 9 | 9 | 2815 |
| 10 | 8 | 3625 |
