# Supplementary material for: Probing the SELEX Process with Next-Generation Sequencing
Source: PLoS One. 2011 Dec 29;6(12):e29604. doi: 10.1371/journal.pone.0029604 (PMC3248438; doi:10.1371/journal.pone.0029604)
Supplement: Table S3 — list of clones, sequenced by Sanger method. (DOC) [file pone.0029604.s004.doc]

| **clone** | **Sequence** | **Consensus** |
| --- | --- | --- |
| 1 | 5'-AAT AGC CCC CTG ACG CAA GGG TTA GAG CAG TAA TCA CGT T | 1 |
| 2 | 5'-GAC TCC GGG ACA TGC CCA TGT CGC AAT GGT GGC CCG GAT T | 2 |
| 3 | 5'-AAT AGC CCC CTG ACG CAA GGG TTA GAG CAG TAA TCA CGT T | 1 |
| 4 | 5'-GAC TCC GGG ACA TGC CCA TGT CGC AAT GGT GGC CCG GAT T | 2 |
| 5 | 5'-GAC TCC GGG ACA TGC CCA TGT CGC AAT GGT GGC CCG GAT T | 2 |
| 6 | 5'-CGG GTT CAC CGT TGC GTC ACG GGG CAA TTC ACC ACT CGC C | 3 |
| 7 | no Insert |  |
| 8 | 5'-ACT TTA GGG CTA ACC TGC GAT CGG TGC GTA CCC CAT GCT C | 4 |
| 9 | 5'-ACC AGA ACG CAC CGA TCG CAG GTT TCT GTG AGC AGG GGG | 5 |
| 10 | 5'-ATC TCC GAT TGC CCC ACG ACG CAG TGG TCG GAG TTA CTT T | 6 |
| 11 | 5'-GAC TCC GGG ACA TGC CCA TGT CGC AAT GGT GGC CCG GAT T | 2 |
| 12 | 5'-GTG TAG GAT CAT CGC CCC CAC GAC GCA GTG GGT GAT CGT A | 7 |
| 13 | 5'-GAC TCC GGG ACA TGC CCA TGT CGC AAT GGT GGC CCG GAT T | 2 |
| 14 | 5'-GAC TCC GGG ACA TGC CCA TGT CGC AAT GGT GGC CCG GAT T | 2 |
| 15 | 5'-CGG GTT CAC CGT TGC GTC ACG GGG CAA TTC ACC ACT CGC C | 3 |
| 16 | 5'-GAC TCC GGG ACA TGC CCA TGT CGC AAT GGT GGC CCG GAT T | 2 |
| 17 | 5'-GTG TAG GAT CAT CGC CCC CAC GAC GCA GTG GGT GAT CGT A | 7 |
| 18 | 5'-ATC TCC GAT TGC CCC ACG ACG CAG TGG TCG GAG ATA CTT T | 6 |
| 19 | 5'-ATC TCC GAT TGC CCC ACG ACG CAG TGG TCG GAG TTA CTT T | 6 |
| 20 | 5'-CGG GTT CAC CGT TGC GTC ACG GGG CAA TTC ACC ACT CGC C | 3 |
| 21 | 5'-GTG TAG GAT CAT CGC CCC CAC GAC GCA GTG GGT GAT CGT A | 7 |
| 22 | 5'-CAC GCG ACC GGC GCA GGT CTG AGG GCA GGC CCA GTA ATA T | 8 |
| 23 | 5'-ATC TCC GAT TGC CCC ACG ACG CAG TGG TCG GAG TTA CTT T | 6 |
| 24 | 5'-ATA AGC ACG CCA GGG ACT ATG ACA CCG TAC CTG CTC T | 9 |
| 25 | 5'-AAT AGC CCC CTG ACG CAA GGG TTA GAG CAG TAA TCA TGG T | 1 |

Supporting Table 3. list of clones, sequenced by Sanger method
