# Supplementary material for: Probing the SELEX Process with Next-Generation Sequencing
Source: PLoS One. 2011 Dec 29;6(12):e29604. doi: 10.1371/journal.pone.0029604 (PMC3248438; doi:10.1371/journal.pone.0029604)
Supplement: Table S4 — list of oligonucleotides used in this study. (DOC) [file pone.0029604.s005.doc]

| **Name** | **Application** | **Sequence** |
| --- | --- | --- |
| Pro5 | Primer | 5'-TGACACCGTACCTGCTCT |
| Pro3 | Primer | 5'-ATAGTCCCTGGCGTGCTT |
| Bank40 | Random library | 5'-TGACACCGTACCTGCTCTNNNNNNNNNNNNNNNNNNNNNNNNNNNNNNNNNNNNNNNNAAGCACGCCAGGGACTATGCTGCAGGCATGCAAG |
| SiA1Pro3 | Index | 5'-CAAGCAGAAGACGGCATACGAATCACGATAGTCCCTGGCGTGCTT |
| SiA2Pro3 | Index | 5'-CAAGCAGAAGACGGCATACGACGATGTATAGTCCCTGGCGTGCTT |
| SiA3Pro3 | Index | 5'-CAAGCAGAAGACGGCATACGATTAGGCATAGTCCCTGGCGTGCTT |
| SiA4Pro3 | Index | 5'-CAAGCAGAAGACGGCATACGATGACCAATAGTCCCTGGCGTGCTT |
| SiA5Pro3 | Index | 5'-CAAGCAGAAGACGGCATACGAACAGTGATAGTCCCTGGCGTGCTT |
| SiA6Pro3 | Index | 5'-CAAGCAGAAGACGGCATACGAGCCAATATAGTCCCTGGCGTGCTT |
| SiA7Pro3 | Index | 5'-CAAGCAGAAGACGGCATACGACAGATCATAGTCCCTGGCGTGCTT |
| SiA8Pro3 | Index | 5'-CAAGCAGAAGACGGCATACGAACTTGAATAGTCCCTGGCGTGCTT |
| SiA9Pro3 | Index | 5'-CAAGCAGAAGACGGCATACGAGATCAGATAGTCCCTGGCGTGCTT |
| SiA10Pro3 | Index | 5'-CAAGCAGAAGACGGCATACGATAGCTTATAGTCCCTGGCGTGCTT |
| SiA11Pro3 | Index | 5'-CAAGCAGAAGACGGCATACGAGGCTACATAGTCCCTGGCGTGCTT |
| SPro5 | adapter | 5'-AATGATACGGCGACCACCGACAGGTTCAGAGTTCTACAGTCCGACATGACACCGTACCTGCTCT |
| control | Neg. control for FLAA | 5'-TGACACCGTACCTGCTCTATGCCTATCATGGGCAACCACGCCAGGGACTAT |

Supporting Table 4. list of oligonucleotides used in this study
